# Establishment of a Rapid Micropropagation System for *Kaempferia parviflora* Wall. Ex Baker: Phytochemical Analysis of Leaf Extracts and Evaluation of Biological Activities

**DOI:** 10.3390/plants10040698

**Published:** 2021-04-05

**Authors:** Han-Yong Park, Kyung-Su Kim, Gunes Ak, Gokhan Zengin, Zoltán Cziáky, József Jekő, Kathalingam Adaikalam, Kihwan Song, Doo-Hwan Kim, Iyyakkannu Sivanesan

**Affiliations:** 1 Department of Bioresource Engineering, Sejong University, 209 Neungdong-ro, Gwangjin-gu, Seoul 05006, Korea; hypark@sejong.ac.kr (H.-Y.P.); khsong@sejong.ac.kr (K.S.); 2 Department of Bioresources and Food Science, Institute of Natural Science and Agriculture, Konkuk University, Seoul 05029, Korea; ks0030@hanmail.net (K.-S.K.); kimdh@konkuk.ac.kr (D.-H.K.); 3 Department of Biology, Faculty of Science, Selcuk University, 42130 Konya, Turkey; akguneselcuk@gmail.com (G.A.); gokhanzengin@selcuk.edu.tr (G.Z.); 4 Agricultural and Molecular Research and Service Institute, University of Nyíregyháza, 4400 Nyíregyháza, Hungary; cziaky.zoltan@nye.hu (Z.C.); jjozsi@gmail.com (J.J.); 5 Millimeter-wave Innovation Technology (MINT) Research Center, Dongguk University-Seoul, Seoul 04620, Korea; kathu@dongguk.edu

**Keywords:** micropropagation, silver oxide nanoparticles, flavonoids, antioxidant activity, enzyme inhibition, *Kaempferia parviflora*

## Abstract

This study aimed to establish a rapid in vitro plant regeneration method from rhizome buds of *Kaempferia parviflora* to obtain the valuable secondary metabolites with antioxidant and enzyme inhibition properties. The disinfection effect of silver oxide nanoparticles (AgO NPs) on rhizome and effects of plant growth regulators on shoot multiplication and subsequent rooting were investigated. Surface sterilization of rhizome buds with sodium hypochlorite was insufficient to control contamination. However, immersing rhizome buds in 100 mg L^−1^ AgO NPs for 60 min eliminated contamination without affecting the survival of explants. The number of shoots (12.2) produced per rhizome bud was higher in Murashige and Skoog (MS) medium containing 8 µM of 6-Benzyladenine (6-BA) and 0.5 µM of Thidiazuron (TDZ) than other treatments. The highest number of roots (24), with a mean root length of 7.8 cm and the maximum shoot length (9.8 cm), were obtained on medium MS with 2 µM of Indole-3-butyric acid (IBA). A survival rate of 98% was attained when plantlets of *K. parviflora* were acclimatized in a growth room. Liquid chromatography with tandem mass spectrometry (LC-MS/MS) was used to determine the chemical profile of *K. parviflora* leaf extracts. Results showed that several biologically active flavonoids reported in rhizomes were also present in leaf tissues of both in vitro cultured and ex vitro (greenhouse-grown) plantlets of *K. parviflora*. We found 40 and 36 compounds in in vitro cultured and ex vitro grown leaf samples, respectively. Greenhouse leaves exhibited more potent antioxidant activities than leaves from in vitro cultures. A higher acetylcholinesterase inhibitory ability was obtained for greenhouse leaves (1.07 mg/mL). However, leaves from in vitro cultures exhibited stronger butyrylcholinesterase inhibitory abilities. These results suggest that leaves of *K. parviflora*, as major byproducts of black ginger cultivation, could be used as valuable alternative sources for extracting bioactive compounds.

## 1. Introduction

Black ginger (*Kaempferia parviflora* Wall. Ex Baker), a medicinal plant of the family Zingiberaceae, is native to Thailand. Black ginger rhizome is used in traditional medicine to cure colic disorder, weakness, lower blood glucose, male impotence, and ulcers [1,2,3]. It possesses antioxidant [4], anti-allergenic [5], anticancer [6], antimicrobial [1], anticholinesterase [7], anti-inflammatory [8], anti-obesity [9], and antimutagenic [10] properties. Phytochemical analysis of black ginger rhizome extracts has confirmed the presence of flavonoids [1,11], methoxyflavones [5,7,10,12,13,14], phenolic glycosides [15,16], and terpenoids [17]. Leaf extract of *Kaempferia galanga* has been reported to exhibit antinociceptive, anti-inflammatory [18], and sedative [19] properties. However, the biological activity and phytochemical profile of *K. parviflora* leaves have not been reported yet.

Multiple uses of *K. parviflora* have necessitated its mass collection as a raw material for pharmaceuticals purposes, leading to the depletion of this wild resource and generating pressure on *K. parviflora* populations [20]. Therefore, a sustainable cultivation method is needed to prevent the depletion of natural populations of *K. parviflora* and meet the growing demand from the pharmaceutical market. *K. parviflora* can be propagated using its rhizomes [20,21]. However, the availability of its rhizome is limited because of its use for extracting commercial metabolites and for preparing *K. parviflora* products. In addition, time (12 months) is required to obtain mature rhizomes [22]. Moreover, the yield and content of bioactive metabolites in *K. parviflora* are often affected by climatic change, abiotic factors, and biotic factors. In this regard, in vitro propagation technologies have been implemented for the mass propagation of various medicinal plants [22,23,24]. The establishment of efficient in vitro cell and plant regeneration techniques is essential for genetic improvement and mass production of valuable *K. parviflora* metabolites. In vitro production of biologically active phytochemicals through cell and organ culture as a reliable method is essential for generating cultures within a short period throughout the year.

In vitro plant regeneration [20,21], microrhizome formation [20,25], and cell suspension-based culturing [26] of *K. parviflora* have been reported. Axillary shoot multiplication has been achieved using terminal shoot buds [21] or rhizomatous buds [20]. The authors used 35.52 µM of 6-BA (6-Benzyladenine) to obtain maximal shoot production [20,21]. Cell suspension of *K. parviflora* can be established the best with liquid Murashige and Skoog [27] (MS) nutrient medium containing 1.0 mg L^−1^ 2,4-D (2,4-dichlorophenoxyacetic acid) [26]. However, the authors did not evaluate phytochemical compositions/contents or biological activities of the shoot, callus, or cell suspension cultures of *K. parviflora* [20,21,26]. Surface sterilization of plant materials obtained from wild-growing plants is essential before in vitro cultivation because bacteria and fungi grow faster in culture media than isolated explants [28]. The elimination of microorganisms attached to the surface of the rhizome is often difficult. In the meantime, the use of multiple chemicals has adverse effects on the survival of explants. Researchers have attempted to use nanoparticles (NPs) such as silver (Ag NPs), zinc (Zn NPs), and titanium dioxide (TiO2 NPs) to solve the contamination issue in plant tissue culture. However, the effectiveness of NPs on the reduction or elimination of contamination depends on their physical properties, concentrations, and exposure time [28].

The goals of this current study were (1) to establish a rapid in vitro plant regeneration method from rhizome buds of *K. parviflora* and (2) phytochemical analysis, antioxidant studies, and enzyme inhibition studies. The disinfection effect of AgO NPs on rhizome and effects of plant growth regulators (PGRs) on shoot multiplication and subsequent rooting were investigated. Gradient reversed-phase ultra-high-performance liquid chromatography (UHPLC) separations with electrospray tandem mass spectrometry (MS/MS) detection (in both positive and negative ion modes) were used for the identification of compounds in leaf extracts. The results showed that several biologically active flavonoids reported in rhizomes were also present in both in vitro cultured and ex vitro (greenhouse)-grown leaf tissues of plantlets of *K. parviflora*. Its leaf extracts also exhibited significant free radical scavenging and enzyme inhibitory abilities in in vitro assays. Therefore, the leaves of *K. parviflora* as major byproducts of black ginger cultivation could be used as valuable alternative sources for extracting bioactive compounds.

## 2. Results and Discussion

### 2.1. In Vitro Micropropagation

#### 2.1.1. Surface Sterilization

In vitro micropropagation is an effective and applicable method for preserving biodiversity and mass production of important plants [29,30,31]. The surface disinfection of in vivo plant materials is a prerequisite for initiating in vitro plant cultures. The initiation of sterile culture using explants obtained from underground parts is tricky because numerous microbes are attached to the surface of explants [22,28]. Previous studies have shown that surface disinfection of rhizome buds with mercuric chloride can reduce contamination in *Kaempferia angustifolia* [32], *K. galanga* [23,24], *K. parviflora* [22], and *Kaempferia rotunda* [23]. In our preliminary experiment, surface sterilization of *K. parviflora* rhizome buds with mercuric chloride yielded about 65% sterile culture. However, it negatively affected the organogenesis and viability of explants. Silver nitrate and antibiotics have been applied to eliminate contamination in a plant in vitro cultures [33]. Several NPs have been reported to have excellent antimicrobial activities [34]. Silver NP is comparatively free of decontaminators adverse effects, with less toxicity profile and good tissue tolerance [35]. It has been reported that Ag NPs can enter microbial cells and induce changes in intracellular structures, nucleic acids, proteins, and lipids, leading to cell death [36]. Recently, it has been shown that Ag NPs can reduce or eliminate microbial contamination in a wide range of plants, such as almond × peach rootstock [35], *Araucaria excelsa* [37], *Capparis decidua* [38], and *Valeriana officinalis* [39]. In this study, rhizome buds of *K. parviflora* were subjected to surface disinfection using AgO NPs. Rhizome buds treated with sodium hypochlorite served as controls. Decontamination and explant survival rates were significantly (*p* = 0.001) affected by AgO NP concentration, exposure time, and their interaction (Table 1).

**Figure 1:**
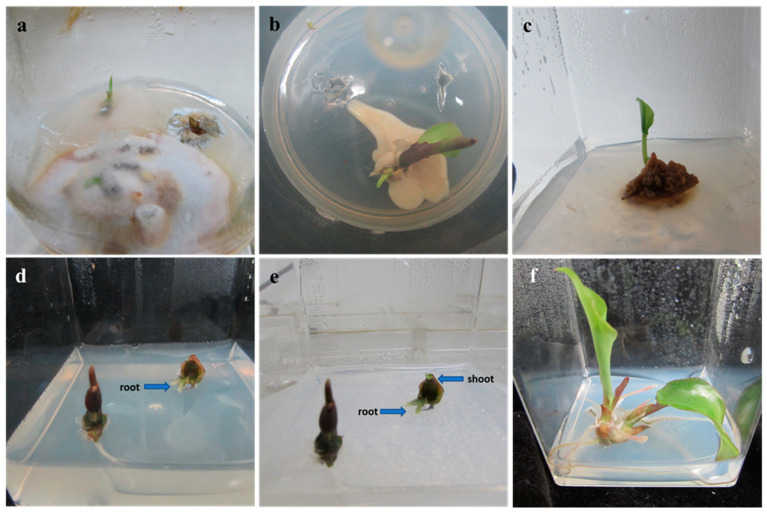
Photograph showing contamination in (**a**) sodium hypochlorite-treated explants, (**b**) 25 mg L^−1^ AgO NPs-treated explants, and (**c**) 50 mg L^−1^ AgO NPs-treated explants. (**d**) Root initiation after 9 days of culture, (**e**) shoot induction after 17 days of culture, (**f**) multiple plantlets regeneration after 42 days of culture.

Surface sterilization of rhizome buds with sodium hypochlorite was insufficient to control the contamination (Figure 1a). However, the soaking of predisinfected rhizome buds in different AgO NPs significantly inhibited contaminants compared to the control. Treating explants with 25 mg L^−1^ or 50 mg L^−1^ AgO NPs was insufficient to control surface contaminants (Figure 1b,c). A high level of AgO NPs (200 mg L^−1^) harmed explants survival (Table 2). It is well known that doses of decontaminators and exposure period can affect the morphogenetic potential and survival of explants [28,38]. Increasing the concentration and duration of exposure of AgO NPs also increased the rate of decontamination. However, the reverse was observed for the survival rate of *K. parviflora* explants (Table 2). With increasing doses, decontamination and survival rates of explants were increased and decreased, respectively. Among the AgO NPs treatments evaluated, immersing rhizome buds for 60 min in 100 mg L^−1^ AgO NPs eliminated contamination without affecting the survival of explants (Table 1). Similar results have been reported for almond × peach rootstock [35], *A. excelsa* [37], *C. decidua* [38], and *V. officinalis* [39].

#### 2.1.2. Shoot Multiplication

Rhizome buds of *K. parviflora* were cultured on MS nutrient medium containing 0–12 µM of cytokinin for shoot multiplication (Table 3). Roots first appeared after 9 days (Figure 1d) and shoots appeared after 17 days (Figure 1e) of cultivation. *K. parviflora* rhizome buds produced multiple plantlets within 42 days (Figure 1f) of cultivation. These rhizome bud explants (22.6%) produced shoots (1.2) and roots (2.3) together after 56 days on a PGR-free medium. The addition of 6-BA, 6-furfuryladenine (6-KN), or Thidiazuron (TDZ) at 1–12 µM increased the rate of regeneration, number of shoots per *K. parviflora* rhizome bud, and number of roots per shoot. Cytokinins are important PGRs that can promote axillary shoot multiplication [29,31,40], somatic embryogenesis [41], and adventitious shoot regeneration [42] in numerous plants. However, the application of cytokinin often adversely affects in vitro rhizogenesis [31]. In this study, simultaneous regeneration of both shoots and roots was attained using medium MS even with a high cytokinin level. Similar results have been disclosed earlier for *K. galanga* [43], *K. parviflora* [21], *Hedychium coronarium* [44,45], *Globba marantina* [46], and *Hosta minor* [40]. Cytokinin, concentration, and cytokinin × concentration interaction had significant effects on the regeneration rate and the number of shoots. Although cytokinin had no significant (*p* = 0.306) effect on the number of roots per shoot, the concentration of cytokinin (*p* = 0.001) and cytokinin × concentration interaction (*p* = 0.028) significantly affected the induction of roots (Table 3).

**Figure 2:**
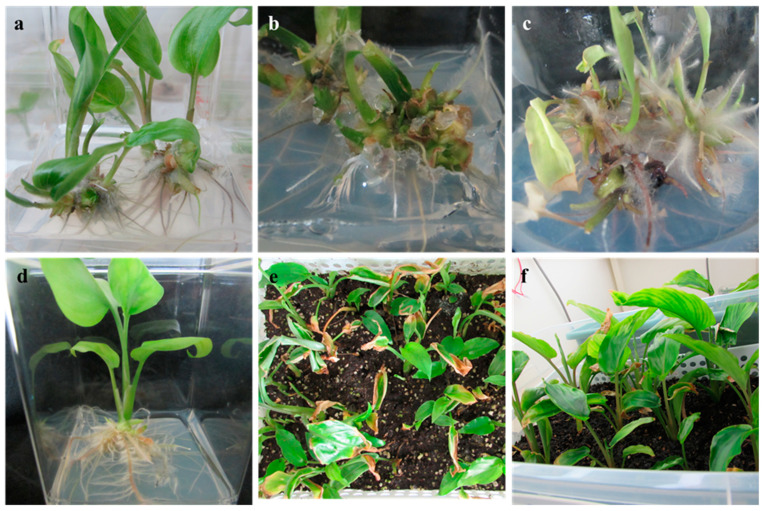
Micropropagation of *Kaempferia parviflora*. (**a**) Rhizome buds cultivated on Murashige and Skoog (MS) nutrient medium with 8 µM 6-BA; (**b**) rhizome buds cultivated on MS nutrient medium with 8 µM 6-BA and 0.5 µM Thidiazuron (TDZ); (**c**) rhizome buds cultivated on MS nutrient medium with 8 µM 6-BA, 0.5 µM TDZ, and 1 µM Naphthalene-1-acetic acid (NAA); (**d**) well-developed plantlet cultivated on nutrient medium MS with 2 µM Indole-3-butyric acid (IBA); (**e**,**f**) acclimatization.

Shoot formation rate, number of shoots per rhizome bud, and number of roots per shoot varied from 34.0% to 78.8%, 1.4 to 6.3, and 2.9 to 5.3, respectively, when the medium MS was added with 1–12 µM of 6-BA. Rhizome buds of *K. parviflora* (78.8%) produced multiple shoots (6.3) and roots (4.6) on medium MS containing 8 µM of 6-BA (Figure 2a, Table 3). Shoots formed on medium MS added with 2 µM of 6-BA developed maximal roots (5.3). Explant response, shoot production, and root production were decreased on medium MS containing 12 µM of 6-BA. In contrast, terminal buds of *K. parviflora* produced a maximum of 7.16 shoots on medium MS added with 35.52 µM of 6-BA [21]. Shooting response, number of shoots per rhizome bud, and number of roots per shoot varied from 23.0% to 61.1%, 1.3 to 3.7, and 1.9 to 6.6, respectively, when the medium MS was added with 1–12 µM of 6-KN. The best shoot formation (61.1%), number of shoots (3.7), and number of roots (6.6) were noticed on medium MS added with 12 µM, 4 µM, and 2 µM of 6-KN, respectively (Table 3). The response of rhizome buds, number of shoots per rhizome bud, and number of roots per shoot varied from 32.7% to 67.8%, 2.6 to 4.7, and 2.1 to 5.0, respectively, when the medium MS was added with 1–12 µM of TDZ. The maximal response (67.8%), number of shoots (4.7), and number of roots (5.0) were noticed for medium MS added with 2 µM of TDZ (Table 3). Regeneration response, shoot production, and root production was found to be meager on medium MS added with 12 µM of TDZ. Detrimental effects of TDZ at a high dose on shoot production have also been disclosed for *K. parviflora* [21]. Among the cytokinins evaluated, 6-BA yielded the best explant response (60.2%), followed by TDZ (48.5%) and 6-KN (43.6%). Advantages of 6-BA on plant regeneration in vitro have been disclosed for *K. parviflora* [20,21] and other Zingiberaceae members such as *Curcuma angustifolia* [47], *H. coronarium* [44], and *K. galanga* [43]. However, a significant difference in the number of roots was not found among cytokinins (Table 4). Among all concentrations (1–12 µM) evaluated, 8 µM produced a higher shooting rate (63.3%) than other levels. However, 6-BA at a concentration of 2 µM, 4 µM, or 8 µM had a similar impact (*p* = 0.05) on *K. parviflora* shoot production (Table 4).

Several works have shown that a combination of PGRs can boost the regeneration of multiple shoots for Zingiberaceae members [43,44,46,47]. Chithra et al. [43] used a combination of 11.4 µM silver nitrate, 8.8 µM 6-BA, and 2.46 µM Indole-3-butyric acid (IBA) to induce maximal axillary buds (8.3) and roots (6.7) for *K. galanga*. Mohanty et al. [44] used a combination of 8.8 µM 6-BA and 2.7 µM NAA to obtain maximal axillary buds (3.6) and roots (4) for *H. coronarium*. Parida et al. [46] used a combination of 14.1 µM 6-KN and 2.7 µM Naphthalene-1-acetic acid (NAA) to induce 9.5 axillary shoots and 4.5 roots for *G. marantina*. Jena et al. [47] used 135.7 µM adenine sulfate, 13.3 µM 6-BA, and 5.7 µM Indole-3-acetic acid (IAA) to obtain higher shoots (14.1) and roots (7.6) for *C. angustifolia*. In the present study, rhizome bud explants were placed on OM (optimal medium: MS plus 8 µM 6-BA) combined with other PGRs (Table 5) to produce roots and shoots within 14 days of cultivation. Supplementation of 2 µM and 4 µM of 6-KN to OM enhanced the explant response (89%) and number of shoots (9.2). Similarly, supplementation of 0.5–2 µM TDZ to OM enhanced the explant response (83.6–97.2%). The number of shoots (12.2) was higher in OM added with 0.5 µM TDZ after 56 days (Figure 2b, Table 5). The addition of 1–4 µM of NAA to OM containing 0.5 µM TDZ resulted in the maximum explant response (100%). However, shoot production was decreased (Figure 2c, Table 5). The number of roots increased as NAA level increased from 1–4 µM. Prathanturarug et al. [21] also reported that NAA and cytokinin (6-BA, TDZ) in combination cannot increase shoot regeneration for *K. parviflora*.

#### 2.1.3. Rooting and Acclimatization

Although rhizome buds of *K. parviflora* developed both shoots and roots on OM alone or in combination with 0.5 µM TDZ, adventitious roots failed to develop lateral roots even after 56 days of cultivation (Figure 2a,b). Several studies have shown that cytokinins have detrimental effects on lateral root induction (reviewed by Jing and Strader [48]). In general, auxin is often included in a rooting medium to induce rhizogenesis of cultured shoots. IBA is a notable auxin that can stimulate rhizogenesis of diverse plant species [29,31,40]. Therefore, shoot buds (4 weeks old) were transferred to basal medium MS added with IBA (0–12 µM) to induce and develop roots. The addition of IBA to rooting medium MS improved the rooting quality (Figure 2d). Medium MS added with 2 µM of IBA resulted in the highest number of roots (24) with a mean root length of 7.8 cm and the maximum shoot length (9.8 cm) (Table 6). Shoot buds of *K. parviflora* on medium MS added with 4 µM of IBA developed longer roots (8.9 cm) than those in other treatments. Plantlets of *K. parviflora* were acclimatized well in a growth room, having a survival rate of 98% (Figure 2e,f).

### 2.2. Phytochemical Compositions

Phenolic compounds are considered as leading contributors to the biological activities of plant extracts. In the present study, we determined total amounts of phenolics and flavonoids in *K. parviflora* extracts. Results are presented in Table 7. Leaves from the greenhouse (18.28 mg GAE/g extract) contained higher phenolics levels than leaves from in vitro cultures (14.07 mg GAE/g extract). However, levels of total flavonoids in leaves from in vitro cultures (1.55 mg RE/g extract) were higher than those from greenhouse ones (0.96 mg RE/g extract). These results indicate that in vitro culture conditions could enhance levels of flavonoids. Previously published papers have also indicated that levels of total flavonoids are changed under in vitro culture conditions [49,50,51]. Krongrawa et al. [52] reported that levels of total phenolics and flavonoids in *K. parviflora* are 17.88–19.07 mg GAE/g and 15.90–16.68 mg QE/g, respectively, after gamma radiation. In addition, Choi et al. [53] reported that the contents of total phenolics in different fractions of *K. parviflora* are 19.48–92.26 mg GAE/g extract. These different levels of total phenolics could be due to different factors, including in vitro culture conditions, extraction methods, and solvents. On the other hand, spectrophotometric measurements have some drawbacks. Most phytochemists do not use them to perform content analysis. For example, recent papers have shown that the Folin–Ciocalteu assay measures reducing power instead of total phenolics content [54]. To obtain more accurate levels of total phenolics, at least one chromatographic technique has been suggested recently [55,56,57]. In this sense, the chemical profile of *K. parviflora* extracts was identified by LC-MS/MS.

Samples were analyzed by UHPLC to obtain chromatographic profiles of more polar portions of extracts known to mainly contain phenolic and flavonoid compounds.

All characterized compounds with their chromatographic data, MS data (retention times, protonated or deprotonated molecular ions, fragment ions), and assigned identities are shown in Table 8 and Table 9. Compounds were numbered by their elution order in a 56-day-old in vitro sample. These same numbers were used in a 90-day-old ex vitro sample. We found 40 and 36 compounds in in vitro and in vivo samples, respectively. Both samples showed a similar chromatographic profile. A wide range of compounds, mainly flavonoids, were characterized.

**Figure 3:**
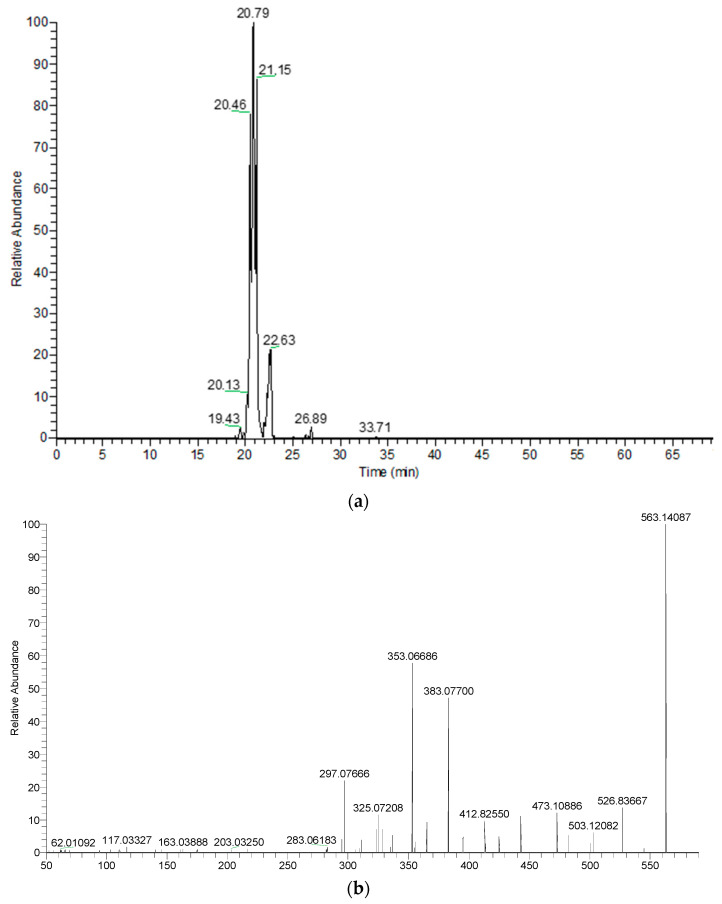
(**a**) Extracted ion chromatogram of apigenin C-hexoside-C-pentoside isomers (*m*/*z*: 563.14087) in negative ion mode (56-day-old in vitro sample). (**b**) MS2 spectrum of apigenin C-hexoside-C-pentoside isomer 1 in negative mode at a retention time of 20.52 min (56-day-old in vitro sample).

**Figure 4:**
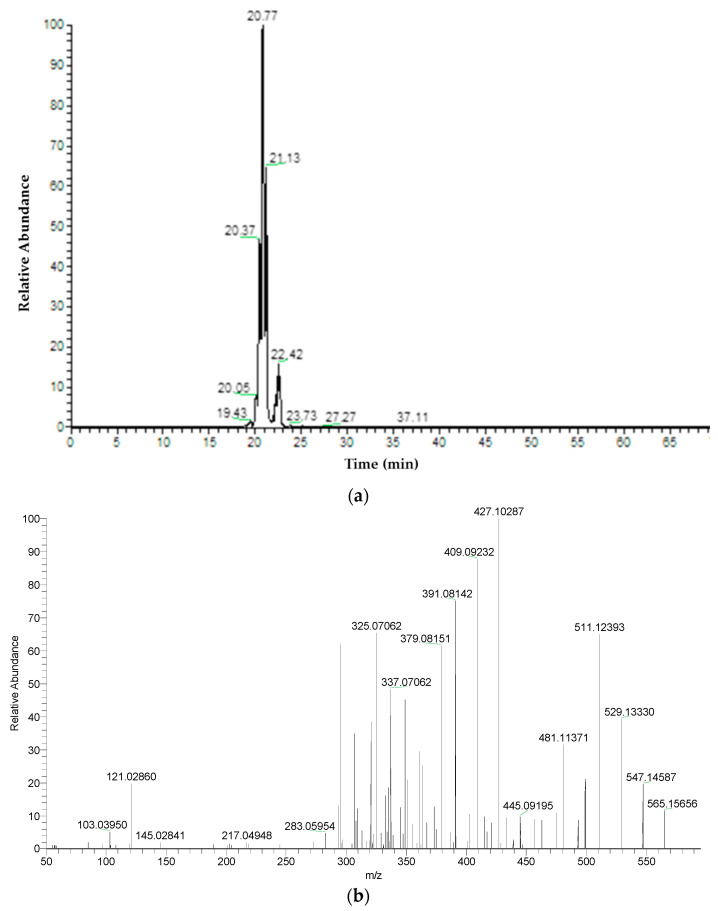
(**a**) Extracted ion chromatogram of apigenin C-hexoside-C-pentoside isomers (*m*/*z*: 565.15574) in positive ion mode (56-day-old in vitro sample). (**b**) MS2 spectrum of apigenin C-hexoside-C-pentoside isomer 1 in positive mode at a retention time of 20.41 min (56-day-old in vitro sample).

The lowest molecular mass component was caffeic acid (1) (rt: 15.19 min, MW: 180.04226). Compound 12 had the highest molecular mass. It was characterized as syringetin-3-O-rutinoside (MW: 654.17960). Compound 2 at rt: 19.38 min was confirmed as vicenin-2 (apigenin 6,8-di-C-glucoside). Characteristic fragments confirmed the substitution of two C-glucosides at positions 6 and 8 in compound 2. Compounds at rt: 20.46 min, 20.79 min, and 21.15 min were identified as apigenin C-hexoside-C-pentoside isomers (4–6) with [M − H]− at *m*/*z* 563.1409, showing ion fragments at *m*/*z* 473.1089 corresponding to [M − H-90]−, *m*/*z* 443.0974 corresponding to [M − H-120]−, *m*/*z* 383.0770 corresponding to [M − H-180]−, and *m*/*z* 353.0669 corresponding to [M − H-120-90]− in the MS/MS spectrum (Figure 3a,b). The positive ion mode was a powerful complementary tool of the negative ion mode for compounds’ structural characterization by electrospray ionization (ESI)-MS/MS. In compound 4–6, more fragment ions were detected in the positive mode (Figure 4a,b).

### 2.3. Antioxidant Ability

To detect the antioxidant potential of *K. parviflora* in the present study, we used six assays. Antioxidant compounds are closely linked to positive effects on human health. They can minimize the negative effects of free radicals that are instable and very active. Scavenging of free radicals (3-ethylbenzothiazoline-6-sulphonic acid) (ABTS) and 2,2-diphenyl-1-picrylhydrazyl (DPPH)) was tested. Results are given as IC_50_ values. As shown in Table 10, *K. parviflora* leaf extracts exhibited low DPPH scavenging abilities, with IC_50_ values >3 mg/mL. Regarding ABTS scavenging abilities, greenhouse leaves exhibited more potent activities than leaves from in vitro cultures. However, these tested extracts had weaker scavenging abilities compared to Trolox (IC_50_: 0.06 mg/mL for DPPH and 0.09 mg/mL for ABTS). Their reducing abilities were evaluated by cupric reducing antioxidant capacity (CUPRAC) and ferric reducing antioxidant power (FRAP) assays. In both assays, greenhouse leaves possessed higher abilities (CUPRAC and FRAP: 2.07 mg/mL and 1.52 mg/mL, respectively). Reducing power assays reflect the electron-donating abilities of plant extracts and antioxidant compounds. Phosphomolybdenum assay is based on Mo (VI) transformation to Mo (V) by antioxidant compounds at an acidic condition. Both leaves samples had weak ability in phosphomolybdenum assays. Their IC_50_ values were higher than 3 mg/mL. In the last assay, the metal chelating abilities of leaf extracts were determined. Results showed that leaves from in vitro cultures had higher metal-chelating abilities, with an average IC_50_ value of 0.59 mg/mL. However, ethylenediaminetetraacetic acid was a better chelator. Several studies have reported antioxidant properties of *K. parviflora* extracts or fractions. For example, Krongrawa et al. [52] demonstrated antioxidant properties of *K. parviflora* extracts using DPPH and FRAP assays. In their study, IC_50_ values ranged from 129.08 µg/mL to 165.26 µg/mL in the DPPH assay. The reducing power in FRAP assay was found to be 11.96–12.48 mg ascorbic acid equivalent/g extract. Thao et al. [4] reported antioxidant abilities for peroxyl radicals and cupric reducing power of *K. parviflora* rhizomes. Choi et al. [53] disclosed that the ethyl acetate fraction of *K. parviflora* has the strongest DPPH, ABTS, and ferric reducing power. As can be seen in earlier papers, few reports are available on the antioxidant properties of *K. parviflora*. Thus, the results of the present study provide valuable scientific knowledge of *K. parviflora*.

### 2.4. Enzyme Inhibitory Effects

Although the world is a healthier place today, humanity still faces global health problems. Several infectious diseases, including polio, Ebola, and smallpox, have been eliminated by some effective treatment strategies over the centuries. However, the prevalence of some noncommunicable diseases is almost epidemic all over the world. For example, about 500 million people are affected by diabetes mellitus [58]. In this sense, we need effective therapeutic tools to overcome the burden. Many studies have demonstrated that enzymes are effective drug targets [59]. According to this approach, inhibition of some clinical enzymes is linked to the alleviated symptoms observed. For example, inhibition of amylase and glucosidase as main hydrolyzing enzymes of carbohydrates can control blood glucose levels after a carbohydrate-rich diet [60]. Thus, enzyme inhibitors are among the most common topics in medical and pharmaceutical areas. Researchers have attempted to use several chemicals for this purpose. However, these chemicals have produced unpleasant effects, including toxicity and gastrointestinal disturbances [61,62]. Taken together, these findings suggest that we need to replace synthetics with safe and effective ones from natural resources.

In the current paper, we tested the enzyme inhibiting properties of *K. parviflora* leaves. Results are given as IC_50_ values (Table 11). The best AChE inhibitory ability was obtained for leaves from greenhouse-grown *K. parviflora* (1.07 mg/mL). However, leaves from in vitro cultures exhibited stronger BChE inhibitory abilities than leaves from the greenhouse. Regarding tyrosinase inhibitory activity, both leaves had some potential, with an average IC_50_ value of 0.71 mg/mL. Finally, the best amylase inhibition ability was obtained for leaves from the greenhouse, with an IC_50_ value of 1.37 mg/mL. However, inhibitor standards were more active than tested extracts in all assays performed. Observed enzyme inhibitory abilities might be explained by the presence of some compounds in these leaf extracts. For example, some phenolic acids such as caffeic [63,64,65] and ferulic acids [66,67,68] have been shown to possess significant inhibitor properties in earlier studies. Again, flavonoids including rutin [69,70] and quercetin [69,71] have been reported as effective enzyme inhibitors. Thus, *K. parviflora* leaves could be useful as sources of natural enzyme inhibitors for pharmaceutical and cosmetic applications.

## 3. Materials and Methods

### 3.1. In Vitro Micropropagation

#### 3.1.1. Synthesis and Characterization of Silver Oxide Nanoparticles (AgO NPs)

Silver oxide nanoparticles were synthesized with the hydrothermal method using polyethylene glycol and silver nitrates purchased from Sigma-Aldrich. For the synthesis of AgO nanoparticles, 25 g of polyethylene glycol (PEG) was dissolved in 1 L of deionized (DI) water and stirred unceasingly for 1 h at 60 °C. After complete dissolution as a homogeneous solution, 1 g of silver nitrate salt was added into the aqueous PEG solution under constant stirring for another 1 h. After a set period, formed AgO nanoparticles were filtered using a membrane filter (0.2 μm, Millipore). These filtered particles were washed several times with DI water. After washing with ethanol, they were then dried in an oven at 60 °C overnight [72]. These dried AgO nanoparticles were characterized by a field-emission scanning electron microscope (FESEM) attached with an energy-dispersive X-ray analysis (EDAX) setup to determine their morphological and composition properties. A Hitachi Ultrahigh Resolution SEM (S-4800) attached with an EDAX module was used for morphological and compositional characterization of silver oxide nanoparticles. For SEM characterization, synthesized particles were spread onto adhesive conductive carbon tapes. The platinum metal was then used to coat these particles.

**Figure 5:**
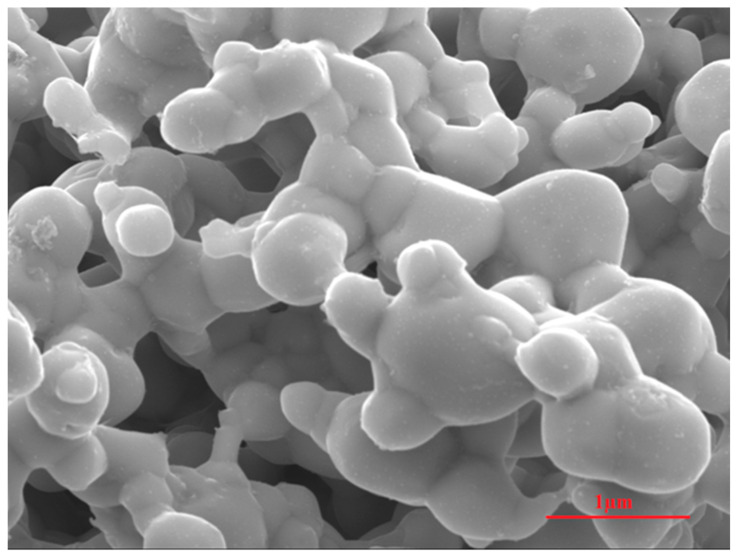
Field-emission scanning electron microscope (FESEM) image of synthesized AgO nanoparticles.

**Figure 6:**
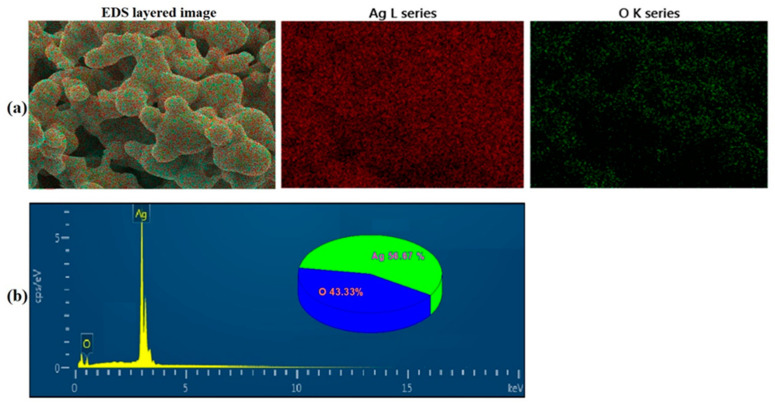
(**a**) Energy-dispersive X-ray (EDX) mapping and (**b**) spectrum of AgO nanoparticles.

SEM images of hydrothermally synthesized silver oxide nanoparticles are presented in Figure 5, showing that these AgO particles were spherical in shape with different sizes due to the highly agglomerated nano-crystallite gains of AgO. The surface of these agglomerated particles clearly showed nanocrystalline grains, confirming the nano-nature of the synthesized AgO. The compositions of these synthesized AgO particles are presented in Figure 6, showing the EDX mapping (Figure 6a) and EDX spectrum (Figure 2b) of the product. EDX mapping displayed a uniform distribution for both Ag and O elements. The composition levels are shown in Figure 6b. The results confirmed a stoichiometric formation of AgO nanoparticles through hydrothermal synthesis.

#### 3.1.2. Plant Materials and Surface Decontamination

Rhizomes of *K. parviflora* harvested from field-grown plants were cleaned under running tap water, planted in plastic trays containing a mixture of autoclaved perlite/peat moss (1:1, *v*/*v*), and kept in a growth room in darkness at 24 ± 1 °C. After 3 weeks, rhizome developing buds were isolated, soaked in detergent solution (0.01%, *v*/*v*) for 5 min, and then thoroughly washed under tap water for 45 min. These rhizome buds were sterilized in sodium hypochlorite (2.5% *v*/*v*) for 12 min and rinsed several times with sterilized distilled water. These rhizome buds were again immersed in 0–200 mg L^−1^ AgO NPs suspension for 30 min, 60 min, or 90 min and rinsed 6 times with sterilized distilled water. These buds were excised from sterilized rhizomes and placed on MS nutrient medium containing 2.0 µM Thidiazuron (TDZ), 3% sucrose, and 0.8% plant agar (pH 5.6–5.8). Cultures were kept at 24 ± 1 °C for 21 days with a 16h/8h light/dark photoperiod (40–45 µmol m^−2^ s^−1^) provided by cool white fluorescent tubes. Experiments were conducted with as a completely randomized design (CRD). In each treatment, 20 rhizome buds were used with 3 replications. All experiments were performed twice. The decontamination rate was recorded at 7 days after incubation, and the survival rate of explants was determined at 3 weeks after incubation.

#### 3.1.3. Shoot Multiplication

Buds were excised from 100 mg L^−1^ Ag2O NPs treated *K. parviflora* rhizomes cultured on MS nutrient medium containing 0–12 µM TDZ, 6-furfuryladenine (6-KN), or 6-BA and 8 µM 6-BA. The basal medium was then combined with 2 µM, 4 µM, and 6 µM 6-KN; 0.5 µM, 1 µM, and 2 µM TDZ; or 0.5 µM TDZ and 1 µM, 2 µM, or 4 µM Naphthalene-1-acetic acid (NAA) to induce multiple shoots. These cultures were kept for 8 weeks at 24 ± 1 °C with a 16 h/8 h light/dark photoperiod (40–45 µmol m^−2^ s^−1^) provided by cool white fluorescent tubes. Experiments were conducted as a CRD (20 rhizome buds were used in each treatment, 3 replications). All experiments were performed twice. Regeneration rate, number of shoots per rhizome bud, and number of roots per induced shoot were assessed after 8 weeks of incubation.

#### 3.1.4. Rooting and Acclimatization

Four-week-old in vitro-induced shoots (≥2–3 cm in height) were obtained from shoot clusters and cultivated on nutrient medium MS containing 0–12 µM Indole-3-butyric acid (IBA) to induce the growth of shoots and roots. After 6 weeks, the number of roots, lengths of roots, and lengths of shoots were recorded. Plantlets were removed from the medium MS containing 2 µM IBA, cleaned with tap water, and transplanted into plastic trays containing sterile soilless substrates composed of 40% peat moss, 30% perlite, and 30% vermiculite based on volume. They were kept in a growth room at 24 ± 1 °C with a 16 h/8 h light/dark photoperiod (90 µmol m^−2^ s^−1^) and irrigated at 3-day intervals. The survival of plants was recorded after 5 weeks. Experiments were conducted as a CRD (50 shoots or plantlets for each treatment with three replications). All experiments were performed twice.

### 3.2. Phytochemical Analysis

#### 3.2.1. Extract Preparation

Leaves of *K. parviflora* were collected from in vitro cultured plantlets (56 days old) and greenhouse-grown plants (90 days old), minced, stored at −70 °C for 12 h, and lyophilized. Dried leaf powder samples of *K. parviflora* (50 mg) were extracted with 80% methanol using an Ultraturrax at 6000 g for 30 min. After filtration, extracts were dried using a rotary vacuum evaporator and kept at 4 °C until further investigation.

#### 3.2.2. Estimation of Total Phenolics Content (TPC) and Flavonoids Content (TFC)

TPC of *K. parviflora* leaf extract was determined using the Folin–Ciocalteu assay described by Slinkard and Singleton [73] and calculated as mg of gallic acid equivalent (GAE). TFC of *K. parviflora* leaf extract was determined using the aluminum chloride (AlCl_3_) technique according to Zengin et al. [74] and expressed as mg of rutin equivalent (RE).

#### 3.2.3. Chemical Characterization

Chromatographic separation was accomplished with a Dionex Ultimate 3000RS UHPLC instrument equipped with a Thermo Accucore C18 (100 mm × 2.1 mm i. d., 2.6 μm) analytical column for separation of compounds. Water (A) and methanol (B) containing 0.1% formic acid were employed as mobile phases. The total run time was 70 min. The elution profile and exact analytical conditions have been published [75]. Electrospray ionization (ESI) was performed in both negative and positive ion modes to obtain more data. Mass spectra were recorded between *m*/*z* 100 and 1500 atomic mass units using a Q-Exactive (Thermo Fisher Scientific, Waltham, MA, USA) Orbitrap mass spectrometer. Chemical constituents were identified by comparison with authentic standards and their MS/MS spectra as well as fragmentation patterns. Peaks and spectra were processed using the TraceFinder software and tentatively identified by comparing their (Rt) retention time and mass spectrum based on reported data and library search.

### 3.3. Biological Activities of K. parviflora Leaf Extracts

#### 3.3.1. Antioxidant Assay

Several antioxidant assays, such as 2,2-azino-bis (3-ethylbenzothiazoline-6-sulphonic acid) (ABTS), 2,2-diphenyl-1-picrylhydrazyl (DPPH), cupric reducing antioxidant capacity (CUPRAC), ferric reducing antioxidant power (FRAP), metal chelating ability (MCA), and phosphomolybdenum (PBD), were carried out to determine the antioxidant potential of *K. parviflora* leaf extract using published methods [76]. Assays were performed in triplicate.

#### 3.3.2. Enzyme Inhibition Assay

Acetylcholinesterase (AChE), amylase, and butylcholinestrase (BChE), and tyrosinase inhibitory activities of *K. parviflora* leaf extract were conducted in triplicates according to procedures described by Uysal et al. [76].

### 3.4. Statistical Analysis

Data were analyzed using SAS version 9.4 (SAS Institute, NC, USA). Significant difference among means was determined by analysis of variance and Duncan’s multiple range test (DMRT).

## 4. Conclusions

Treatment of *K. parviflora* rhizome buds with AgO NPs solution resulted in excellent surface sterilization. Among all cytokinins (6-BA, 6-KN, TDZ) and their concentrations (1–12 µM) evaluated, 8 µM of 6-BA yielded the best explant response. The supplementation of 6-KN (2 µM and 4 µM) or TDZ (0.5–2 µM) to medium MS containing 8 µM 6-BA enhanced the explant response and the number of shoots. The addition of IBA to rooting medium MS improved rooting quality. Higher levels of phenolics and flavonoids were found in leaves from the greenhouse and in vitro cultures, respectively. Leaf extracts exhibited free radical scavenging and enzyme inhibitory activities in in vitro assays. Phytochemical and biological activities of *K. parviflora* leaf extracts are reported in this study for the first time. Further studies are needed to quantify individual flavonoids in leaf tissues. The micropropagation protocol optimized in the current study can be used for mass-clonal propagation of *K. parviflora*.

## Figures and Tables

**Table 1:** Effect of Ag nanoparticles (NPs) on decontamination and survival of rhizome bud explants of *K. parviflora* after 3 weeks of incubation.

Ag NPs (mg L^−1^)	Duration (min)	Decontamination (%)	Explant Survival (%)
0 (Control)		15.2 ± 3.3 j	100 ± 0.0 a
25	30	23.7 ± 4.6 i	100 ± 0.0 a
50		44.2 ± 3.3 f	100 ± 0.0 a
100		64.6 ± 4.9 c	100 ± 0.0 a
200		94.9 ± 3.1 b	73.7 ± 2.6 d
25	60	31.1 ± 4.6 h	100 ± 0.0 a
50		49.7 ± 3.9 e	100 ± 0.0 a
100		100 ± 0.0 a	100 ± 0.0 a
200		100 ± 0.0 a	65.2 ± 3.6 f
25	90	38.1 ± 3.3 g	91.7 ± 2.5 b
50		56.7 ± 4.8 d	85.2 ± 2.9 c
100		100 ± 0.0 a	67.8 ± 3.1 e
200		100 ± 0.0 a	44.3 ± 2.5 g
Mean		62.93	86.7
R-Square Coefficient of variation		0.9896	0.9889
		5.34	2.28
		F-value	
F-test	Conc	27086.7	1775.4
	Duration	251.4	1149.6
	Conc * Duration	53.4	89.0
		*p*-value	
	Conc	0.001	0.001
	Duration	0.001	0.001
	Conc * Duration	0.001	0.001

Means ± SDs, followed by the same letters within a column, were not significantly different *p* < 0.05 by Duncan’s multiple range test (DMRT).

**Table 2:** Effect of concentrations of AgO NPs and exposure time on decontamination and survival of *K. parviflora* rhizome bud explants.

Factors	Decontamination (%)	Explant Survival (%)
25 mg L^−1^	30.9 d	97.2 a
50 mg L^−1^	50.2 c	95.1 b
100 mg L^−1^	88.2 b	89.3 c
200 mg L^−1^	98.3 a	61.1 d
LSD	1.82	1.11
30 min	56.8 c	93.4 a
60 min	70.2 b	91.3 b
90 min	73.7 a	72.3 c
LSD	1.58	0.96

Means within a column, followed by the same letters within a column, were not significantly different *p* < 0.05 by DMRT.

**Table 3:** Effect of cytokinins on shoot multiplication from rhizome bud explants of *K. parviflora* after 8 weeks of incubation.

Cytokinin	Conc (µM)	Response (%)	No. of Shoots/Explant	No. of Roots/Shoot
Control (MS)	0	22.6 ± 2.2 k	1.2 ± 0.4 h	2.3 ± 0.9 gh
6-BA	1	34.0 ± 3.0 i	1.4 ± 0.5 gh	4.3 ± 1.3 b–f
	2	53.9 ± 2.7 f	2.6 ± 0.9 efg	5.3 ± 1.0 b
	4	64.1 ± 2.9 c	4.2 ± 1.6 bc	3.6 ± 1.0 c–f
	8	78.8 ± 3.1 a	6.3 ± 1.6 a	4.6 ± 1.0 bcde
	12	70.2 ± 4.7 b	3.1 ± 1.1 cdef	2.9 ± 0.9 fgh
6-KN	1	23.0 ± 1.8 k	1.3 ± 0.5 h	3.2 ± 1.2 d–f
	2	29.4 ± 2.9 j	2.8 ± 1.2 def	6.6 ± 1.6 a
	4	46.6 ± 3.1 g	3.7 ± 1.3 bcde	4.8 ± 1.5 bc
	8	57.7 ± 2.9 e	2.3 ± 1.0 fgh	3.8 ± 1.3 c–f
	12	61.1 ± 3.7 d	3.4 ± 1.1 cdef	1.9 ± 0.6 h
TDZ	1	39.7 ± 2.7 h	2.9 ± 0.8 def	3.1 ± 1.5 efgh
	2	67.8 ± 2.3 b	4.7 ± 1.3 b	5.0 ± 1.9 bc
	4	49.2 ± 2.8 g	3.8 ± 1.0 bcd	4.7 ± 2.3 bcd
	8	53.3 ± 2.7 f	3.0 ± 1.5 def	3.7 ± 1.5 c–f
	12	32.7 ± 1.6 i	2.6 ± 0.5 efg	2.1 ± 1.3 h
Mean		49.0	3.08	3.87
R-Square		0.9741	0.5997	0.4839
Coefficient of variation		5.93	35.68	35.30
		F-value		
F-test	Cyto	378.49	6.72	1.20
	Conc	405.83	14.33	20.83
	Cyto * Conc	192.41	10.61	2.26
		*p*-value		
	Cyto	0.001	0.002	0.306
	Conc	0.001	0.001	0.001
	Cyto * Conc	0.001	0.001	0.028

Means ± SDs, followed by the same letters within a column, were not significantly different *p* < 0.05 by DMRT.

**Table 4:** Effect of cytokinin types and their concentration on shoot multiplication from rhizome bud explants of *K. parviflora*.

Factors	Response (%)	No. of Shoots/Explant	No. of Roots/Shoot
6-BA	60.2 a	3.5 a	4.13 a
6-KN	43.6 b	2.7 b	4.07 a
TDZ	48.5 c	3.4 a	3.71 a
LSD	1.23	0.47	0.58
1 µM	32.2 d	1.9 c	3.6 c
2 µM	50.4 c	3.3 ab	5.7 a
4 µM	53.3 b	3.9 a	4.3 b
8 µM	63.3 a	3.9 a	4.0 bc
12 µM	54.7 b	3.0 b	2.3 d
LSD	1.59	0.61	0.75

Means within a column, followed by the same letters within a column, were not significantly different *p* < 0.05 by DMRT.

**Table 5:** Effect of combinations of PGRs on shoot multiplication from rhizome bud explants of *K. parviflora* after 8 weeks of incubation.

PGRs (µM)				Response (%)	No. of Shoots/Explant	No. of Roots/Shoot
6-BA	6-KN	TDZ	NAA			
0	0	0	0	22.6 ± 2.2 g	1.2 ± 0.4 g	2.3 ± 0.9 e
8	2	0	0	89.0 ± 3.7 c	7.8 ± 1.1 cde	5.3 ± 1.4 cd
8	4	0	0	86.1 ± 5.2 d	9.2 ± 1.3 b	4.2 ± 1.0 d
8	6	0	0	76.2 ± 2.2 f	6.2 ± 1.4 f	2.8 ± 0.7 e
8	0	0.5	0	97.2 ± 1.8 b	12.2 ± 1.8 a	4.3 ± 1.2 d
8	0	1	0	90.9 ± 2.5 c	8.1 ± 1.3 bcd	2.7 ± 1.0 e
8	0	2	0	83.6 ± 2.8 e	6.4 ± 1.7 ef	3.0 ± 1.1 e
8	0	0.5	1	100 ± 0.0 a	8.8 ± 1.6 bc	6.3 ± 1.4 c
8	0	0.5	2	100 ± 0.0 a	6.8 ± 1.5 def	8.1 ± 1.2 b
8	0	0.5	4	100 ± 0.0 a	5.9 ± 1.2 f	9.7 ± 1.4 a
Mean				84.6	7.3	4.9
R-Square				0.9878	0.8113	0.8255
Coefficient of variation				3.07	18.89	23.59
F-value				719.82	38.22	42.07

Means ± SDs, followed by the same letters within a column, were not significantly different *p* < 0.05 by DMRT.

**Table 6:** Effect of IBA on in vitro rooting of *K. parviflora* after 6 weeks of cultivation.

IBA (µM)	Number of Roots/Shoot	Root Length (cm)	Shoot Length (cm)
0	6.9 ± 1.5 f	4.7 ± 1.3 d	5.8 ± 0.5 c
1	11.0 ± 1.7 d	5.6 ± 0.4 c	6.1 ± 0.4 c
2	24.0 ± 2.5 a	7.8 ± 0.5 b	9.8 ± 0.5 a
4	15.8 ± 2.4 b	8.9 ± 0.5 a	6.6 ± 0.4 b
8	13.2 ± 1.5 c	5.8 ± 0.5 c	4.4 ± 0.3 e
12	8.9 ± 1.1 e	3.2 ± 0.7 e	5.3 ± 0.4 d
Mean	13.29	5.99	6.3
R-Square	0.9096	0.8911	0.9499
Coefficient of variation	14.02	11.69	6.45
F-Value	96.55	78.53	182.02

Means ± SDs, followed by the same letters within a column, were not significantly different *p* < 0.05 by DMRT.

**Table 7:** Total phenolic and flavonoid contents in the tested extracts.

Sources of Leaves	Total Phenolic Content (mg GAE/g)	Total Flavonoid Content (mg RE/g)
In vitro cultures	14.07 ± 0.09	1.55 ± 0.07
The greenhouse	18.28 ± 0.20	0.96 ± 0.06

**Table 8:** Chemical composition of the black ginger leaves from in vitro cultures.

No.	Name	Formula	Rt	[M + H]^+^	[M − H]^−^	Fragment 1	Fragment 2	Fragment 3	Fragment 4	Fragment 5	Reference
1	Caffeic acid	C_9_H_8_O_4_	15.22		179.03444	135.0438	107.0492				
2	Vicenin-2 (Apigenin-6,8-di-C-glucoside)	C_27_H_30_O_15_	19.39	595.16630		577.1575	559.1442	457.1131	325.0707	295.0601	
3 ^1^	Ferulic acid	C_10_H_10_O_4_	19.94		193.05009	178.0261	149.0600	137.0226	134.0360	121.0278	
4	Apigenin-C-hexoside-C-pentoside isomer 1	C_26_H_28_O_14_	20.37	565.15574		547.1459	511.1239	427.1029	409.0923	295.0602	
5	Apigenin-C-hexoside-C-pentoside isomer 2	C_26_H_28_O_14_	20.77	565.15574		547.1455	511.1242	427.1030	379.0814	295.0602	
6	Apigenin-C-hexoside-C-pentoside isomer 3	C_26_H_28_O_14_	21.13	565.15574		547.1458	511.1236	469.1133	379.0813	295.0602	
7 ^1^	Rutin (Quercetin-3-O-rutinoside)	C_27_H_30_O_16_	23.54	611.16122		465.1043	303.0499	129.0549	85.0289	71.0497	[15]
8	Lumichrome	C_12_H_10_N_4_O_2_	24.45	243.08821		216.0769	200.0820	198.0665	172.0870	145.0761	
9 ^1^	Cosmosiin (Apigenin-7-O-glucoside)	C_21_H_20_O_10_	24.50	433.11347		271.0601	153.0185	119.0493			
10	Isorhamnetin-3-O-rutinoside (Narcissin)	C_28_H_32_O_16_	25.55		623.16122	315.0513	314.0432	299.0202	271.0243	243.0300	[15]
11	Tamarixetin-3-O-rutinoside	C_28_H_32_O_16_	25.74		623.16122	315.0513	314.0435	299.0198	271.0250	243.0294	[16]
12	Syringetin-3-O-rutinoside	C_29_H_34_O_17_	25.96		653.17178	345.0616	344.0539	329.0303	315.0151	301.0363	[16]
13	Acacetin-7-O-glucoside (Tilianin)	C_22_H_22_O_10_	29.06	447.12913		285.0757	270.0523	269.0444	242.0579		[16]
14	Methoxy-trihydroxy(iso)flavanone	C_16_H_14_O_6_	30.20	303.08687		193.0499	167.0340	163.0390	145.0285		
15 ^1^	Apigenin (4’,5,7-Trihydroxyflavone)	C_15_H_10_O_5_	30.29		269.04500	225.0547	201.0548	151.0026	149.0233	117.0331	
16	Isokaempferide (3-Methoxy-4’,5,7-trihydroxyflavone)	C_16_H_12_O_6_	30.97		299.05556	284.0329	256.0371	255.0297	227.0342		
17	Undecanedioic acid	C_11_H_20_O_4_	31.36		215.12834	197.1176	153.1272	125.0955	57.0331		
18	3,4’,5,7-Tetramethoxyflavone or 3’,4’,5,7-Tetramethoxyflavone	C_19_H_18_O_6_	32.44	343.11817		328.0942	327.0862	314.0793	313.0707	285.0765	[11]
19	Pinocembrin (5,7-Dihydroxyflavanone)	C_15_H_12_O_4_	32.77		255.06573	213.0547	151.0023	145.0645	107.0124	83.0122	
20	3,3’,4’,5,7-Pentamethoxyflavone	C_20_H_20_O_7_	32.92	373.12873		358.1046	357.0968	343.0810	327.0863	312.0990	[11]
21	Kaempferide (4’-Methoxy-3,5,7-trihydroxyflavone)	C_16_H_12_O_6_	33.09		299.05556	284.0328	256.0378	227.0340	151.0030		
22	Dihydroxy-methoxy(iso)flavone-O-acetylhexoside	C_24_H_24_O_11_	33.21	489.13969		285.0758	270.0522	269.0442	242.0564		
23	Dimethoxy-trihydroxy(iso)flavone	C_17_H_14_O_7_	33.35		329.06613	314.0434	299.0198	271.0249	227.0338		
24	Dodecanedioic acid	C_12_H_22_O_4_	33.81		229.14399	211.1334	167.1433				
25	5,7-Dimethoxyflavanone	C_17_H_16_O_4_	33.82	285.11268		181.0497	166.0261	138.0317	131.0494	103.0548	[15]
26 ^1^	Chrysin (5,7-Dihydroxyflavone)	C_15_H_10_O_4_	33.85	255.06573		209.0595	153.0184	129.0340	103.0547	67.0185	
27	5,7-Dimethoxyflavone	C_17_H_14_O_4_	34.13	283.09704		268.0729	267.0652	239.0703	238.0622	225.0538	[11]
28 ^1^	Galangin (3,5,7-Trihydroxyflavone)	C_15_H_10_O_5_	34.75	271.06065		242.0572	215.0701	165.0181	153.0184	105.0336	
29	4’,5,7-Trimethoxyflavone	C_18_H_16_O_5_	34.81	313.10760		298.0837	297.0761	269.0809	255.0649	227.0711	[11]
30	3,5,7-Trimethoxyflavone	C_18_H_16_O_5_	34.98	313.10760		298.0836	297.0758	280.0729	279.0652	252.0778	[11]
31	Dihydroxy-methoxy(iso)flavone	C_16_H_12_O_5_	35.05		283.06065	268.0375	267.0294	239.0344	211.0393		
32	Ayanin (3’,5-Dihydroxy-3,4’,7-trimethoxyflavone)	C_18_H_16_O_7_	35.20	345.09743		330.0733	329.0661	315.0499	287.0551	259.0602	[15]
33	3,4’,5,7-Tetramethoxyflavone or 3’,4’,5,7-Tetramethoxyflavone	C_19_H_18_O_6_	35.44	343.11817		328.0940	327.0862	310.0837	285.0760	282.0886	[11]
34	Dihydroxy-dimethoxy(iso)flavone	C_17_H_14_O_6_	35.60	315.08686		300.0628	299.0548	272.0680	271.0602	257.0445	
35	Retusin (5-Hydroxy-3,3’,4’,7-tetramethoxyflavone)	C_19_H_18_O_7_	37.10	359.11308		344.0890	343.0812	329.0655	301.0706		[11]
36	Pinostrobin (5-Hydroxy-7-methoxyflavanone)	C_16_H_14_O_4_	37.14	271.09704		229.0864	173.0599	167.0339	131.0494	103.0547	[15]
37	Tectochrysin (5-Hydroxy-7-methoxyflavone)	C_16_H_12_O_4_	38.08	269.08138		254.0573	226.0624	167.0338			[11]
38	4’,7-Dimethoxy-5-hydroxyflavone	C_17_H_14_O_5_	38.75	299.09195		284.0678	256.0728				[11]
39	5-Hydroxy-3,7-dimethoxyflavone	C_17_H_14_O_5_	38.94	299.09195		284.0678	283.0601	256.0728	255.0649	241.0496	[11]
40	5-Hydroxy-3,4’,7-trimethoxyflavone	C_18_H_16_O_6_	39.39	329.10252		314.0784	313.0707	299.0552	285.0756	271.0598	[11]

^1^ Confirmed by standard.

**Table 9:** Chemical composition of the black ginger leaves from greenhouse.

No.	Name	Formula	Rt	[M + H]^+^	[M − H]^−^	Fragment 1	Fragment 2	Fragment 3	Fragment 4	Fragment 5	Reference
1	Caffeic acid	C_9_H_8_O_4_	15.19		179.03444	135.0438	107.0488				
2	Vicenin-2 (Apigenin-6,8-di-C-glucoside)	C_27_H_30_O_15_	19.38	595.16630		577.1556	559.1453	457.1132	325.0707	295.0602	
3 ^1^	Ferulic acid	C_10_H_10_O_4_	19.95		193.05009	178.0261	149.0596	137.0231	134.0360	121.0278	
4	Apigenin-C-hexoside-C-pentoside isomer 1	C_26_H_28_O_14_	20.43	565.15574		547.1460	511.1242	427.1027	409.0920	295.0602	
5	Apigenin-C-hexoside-C-pentoside isomer 2	C_26_H_28_O_14_	20.78	565.15574		547.1451	511.1239	427.1030	379.0813	295.0601	
6	Apigenin-C-hexoside-C-pentoside isomer 3	C_26_H_28_O_14_	21.14	565.15574		547.1455	511.1245	469.1141	379.0813	295.0602	
7 ^1^	Rutin (Quercetin-3-O-rutinoside)	C_27_H_30_O_16_	23.53	611.16122		465.1033	303.0499	129.0549	85.0290	71.0498	[15]
8	Lumichrome	C_12_H_10_N_4_O_2_	24.46	243.08821		216.0767	200.0819	198.0662	172.0869	145.0760	
10	Isorhamnetin-3-O-rutinoside (Narcissin)	C_28_H_32_O_16_	25.55		623.16122	315.0512	314.0433	299.0203	271.0254	243.0298	[15]
11	Tamarixetin-3-O-rutinoside	C_28_H_32_O_16_	25.74		623.16122	315.0512	314.0440	299.0192	271.0250	243.0288	[16]
12	Syringetin-3-O-rutinoside	C_29_H_34_O_17_	25.97		653.17178	345.0618	344.0536	329.0306	315.0142	301.0355	[16]
13	Acacetin-7-O-glucoside (Tilianin)	C_22_H_22_O_10_	29.06	447.12913		285.0757	270.0522	269.0443	242.0567		[16]
15 ^1^	Apigenin (4’,5,7-Trihydroxyflavone)	C_15_H_10_O_5_	30.29		269.04500	225.0555	201.0546	151.0019	149.0230	117.0330	
41	Dihydroxy-methoxy(iso)flavone isomer 1	C_16_H_12_O_5_	30.85		283.06065	268.0377	240.0423	239.0346	211.0394		
16	Isokaempferide (3-Methoxy-4’,5,7-trihydroxyflavone)	C_16_H_12_O_6_	30.97		299.05556	284.0328	256.0379	255.0297	227.0341		
17	Undecanedioic acid	C_11_H_20_O_4_	31.37		215.12834	197.1175	153.1272	125.0959	57.0331		
18	3,4’,5,7-Tetramethoxyflavone or 3’,4’,5,7-Tetramethoxyflavone	C_19_H_18_O_6_	32.45	343.11817		328.0943	327.0863	314.0783	313.0707	285.0760	[11]
19	Pinocembrin (5,7-Dihydroxyflavanone)	C_15_H_12_O_4_	32.78		255.06573	213.0556	151.0024	145.0649	107.0121	83.0123	
20	3,3’,4’,5,7-Pentamethoxyflavone	C_20_H_20_O_7_	32.93	373.12873		358.1045	357.0966	343.0811	327.0863	312.0991	[11]
22	Dihydroxy-methoxy(iso)flavone-O-acetylhexoside	C_24_H_24_O_11_	33.20	489.13969		285.0757	270.0523	269.0440	242.0566		
24	Dodecanedioic acid	C_12_H_22_O_4_	33.81		229.14399	211.1332	167.1425				
25	5,7-Dimethoxyflavanone	C_17_H_16_O_4_	33.82	285.11268		181.0496	166.0258	138.0316	131.0493	103.0545	[15]
26 ^1^	Chrysin (5,7-Dihydroxyflavone)	C_15_H_10_O_4_	33.85	255.06573		209.0596	153.0182	129.0341	103.0546	67.0185	
27	5,7-Dimethoxyflavone	C_17_H_14_O_4_	34.14	283.09704		268.0730	267.0650	239.0702	238.0626	225.0549	[11]
28 ^1^	Galangin (3,5,7-Trihydroxyflavone)	C_15_H_10_O_5_	34.76	271.06065		242.0576	215.0700	165.0183	153.0182	105.0339	
29	4’,5,7-Trimethoxyflavone	C_18_H_16_O_5_	34.82	313.10760		298.0836	297.0761	269.0808	255.0662	227.0694	[11]
30	3,5,7-Trimethoxyflavone	C_18_H_16_O_5_	35.00	313.10760		298.0839	297.0757	280.0730	279.0654	252.0782	[11]
31	Dihydroxy-methoxy(iso)flavone isomer 2	C_16_H_12_O_5_	35.03		283.06065	268.0377	267.0294	239.0345	211.0394		
32	Ayanin (3’,5-Dihydroxy-3,4’,7-trimethoxyflavone)	C_18_H_16_O_7_	35.21	345.09743		330.0733	329.0653	315.0499	287.0549	259.0601	[15]
33	3,4’,5,7-Tetramethoxyflavone or 3’,4’,5,7-Tetramethoxyflavone	C_19_H_18_O_6_	35.45	343.11817		328.0944	327.0863	310.0835	285.0754	282.0886	[11]
34	Dihydroxy-dimethoxy(iso)flavone	C_17_H_14_O_6_	35.60	315.08686		300.0629	299.0552	272.0679	271.0602	257.0436	
35	Retusin (5-Hydroxy-3,3’,4’,7-tetramethoxyflavone)	C_19_H_18_O_7_	37.10	359.11308		344.0890	343.0815	329.0656	301.0706		[11]
36	Pinostrobin (5-Hydroxy-7-methoxyflavanone)	C_16_H_14_O_4_	37.14	271.09704		229.0859	173.0598	167.0339	131.0494	103.0546	[15]
37	Tectochrysin (5-Hydroxy-7-methoxyflavone)	C_16_H_12_O_4_	38.09	269.08138		254.0571	226.0626	167.0342			[11]
39	5-Hydroxy-3,7-dimethoxyflavone	C_17_H_14_O_5_	38.94	299.09195		284.0680	283.0602	256.0729	255.0650	241.0494	[11]
40	5-Hydroxy-3,4’,7-trimethoxyflavone	C_18_H_16_O_6_	39.38	329.10252		314.0784	313.0707	299.0550	285.0757	271.0602	[11]

^1^ Confirmed by standard.

**Table 10:** Antioxidant properties of the tested samples (IC_50_ (mg /mL)).

Sources of Leaves and Standards	DPPH	ABTS	CUPRAC	FRAP	PBD	Chelating
In vitro cultures	>3 b	2.99 ± 0.20 c	2.82 ± 0.03 c	1.94 ± 0.01 c	>3 b	0.59 ± 0.01 b
The greenhouse	>3 b	2.20 ± 0.05 b	2.07 ± 0.01 b	1.52 ± 0.02 b	>3 b	0.70 ± 0.09 c
Trolox	0.06 ± 0.01 a	0.09 ± 0.01 a	0.11 ± 0.01 a	0.04 ± 0.01 a	0.52 ± 0.02 a	nt
EDTA	nt	nt	nt	nt	nt	0.02 ± 0.001 a

nt: Not tested. PBD: Phosphomolybdenum. Means ± SDs, followed by the same letters within a column, were not significantly different *p* < 0.05 by DMRT.

**Table 11:** Enzyme inhibitory properties of tested samples (IC_50_ (mg /mL)).

Sources of Leaves and Standards	AChE	BChE	Tyrosinase	Amylase
In vitro cultures	1.15 ± 0.04 b	0.95 ± 0.10 c	0.71 ± 0.01 b	1.41 ± 0.01 b
The greenhouse	1.07 ± 0.10 b	1.67 ± 0.11 b	0.71 ± 0.01 b	1.37 ± 0.05 b
Galantamine	0.003 ± 0.001 a	0.007 ± 0.002 a	nt	nt
Kojic acid	nt	nt	0.08 ± 0.001 a	nt
Acarbose	nt	nt	nt	0.68 ± 0.01 a

nt: Not tested. Means ± SDs, followed by the same letters within a column, were not significantly different *p* < 0.05 by DMRT.
